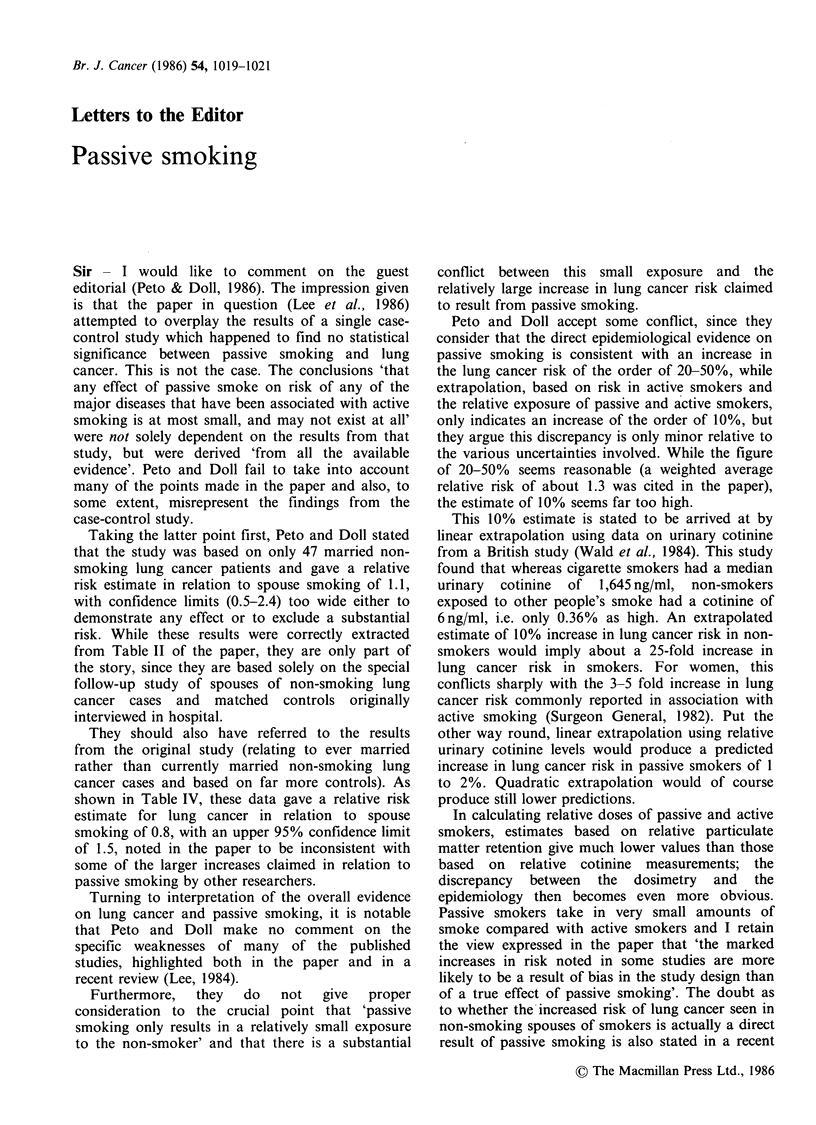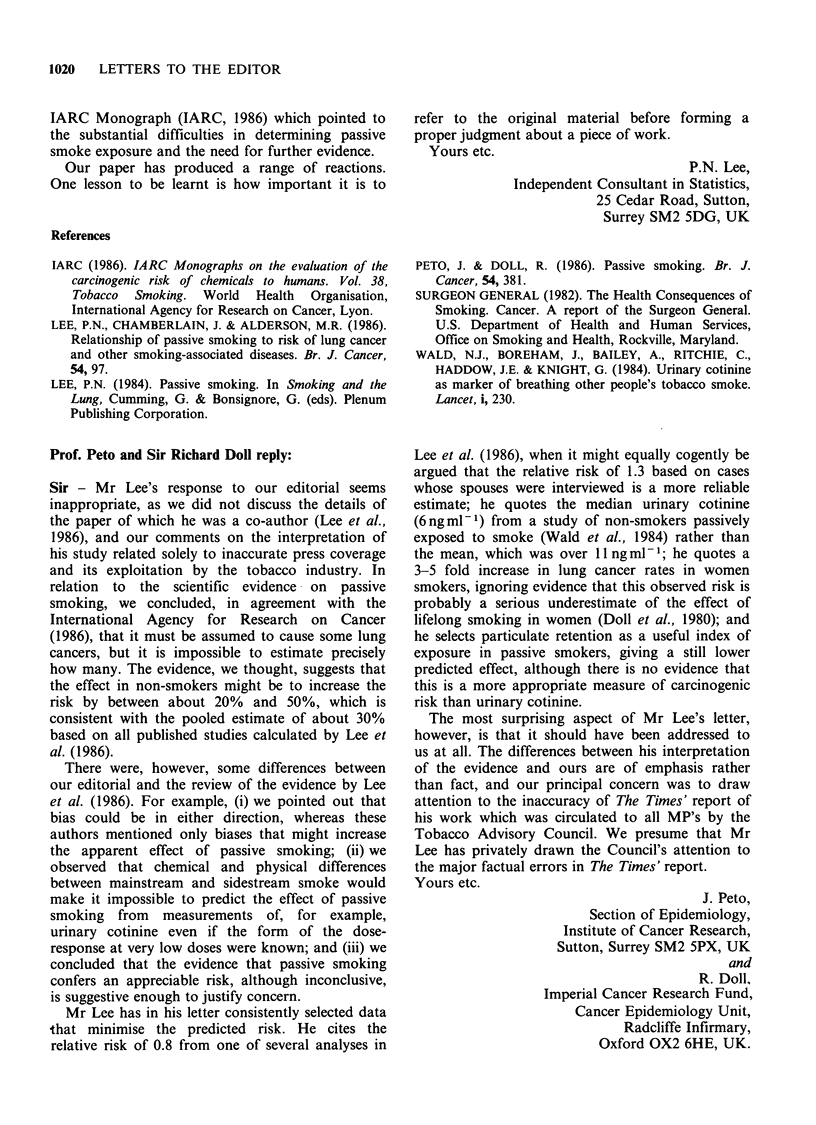# Passive smoking.

**DOI:** 10.1038/bjc.1986.275

**Published:** 1986-12

**Authors:** P. N. Lee


*Br. J. Cancer* (1986) **54**, 1019–1021

### Letters to the Editor

### Passive smoking

**Sir** – I would like to comment on the guest editorial (Peto & Doll, 1986). The impression given is that the paper in question (Lee *et al.*, 1986) attempted to overplay the results of a single case-control study which happened to find no statistical significance between passive smoking and lung cancer. This is not the case. The conclusions 'that any effect of passive smoke on risk of any of the major diseases that have been associated with active smoking is at most small, and may not exist at all' were *not* solely dependent on the results from that study, but were derived 'from all the available evidence'. Peto and Doll fail to take into account many of the points made in the paper and also, to some extent, misrepresent the findings from the case-control study.

Taking the latter point first, Peto and Doll stated that the study was based on only 47 married non-smoking lung cancer patients and gave a relative risk estimate in relation to spouse smoking of 1.1, with confidence limits (0.5–2.4) too wide either to demonstrate any effect or to exclude a substantial risk. While these results were correctly extracted from Table II of the paper, they are only part of the story, since they are based solely on the special follow-up study of spouses of non-smoking lung cancer cases and matched controls originally interviewed in hospital.

They should also have referred to the results from the original study (relating to ever married rather than currently married non-smoking lung cancer cases and based on far more controls). As shown in Table IV, these data gave a relative risk estimate for lung cancer in relation to spouse smoking of 0.8, with an upper 95% confidence limit of 1.5, noted in the paper to be inconsistent with some of the larger increases claimed in relation to passive smoking by other researchers.

Turning to interpretation of the overall evidence on lung cancer and passive smoking, it is notable that Peto and Doll make no comment on the specific weaknesses of many of the published studies, highlighted both in the paper and in a recent review (Lee, 1984).

Furthermore, they do not give proper consideration to the crucial point that 'passive smoking only results in a relatively small exposure to the non-smoker' and that there is a substantial conflict between this small exposure and the relatively large increase in lung cancer risk claimed to result from passive smoking.

Peto and Doll accept some conflict, since they consider that the direct epidemiological evidence on passive smoking is consistent with an increase in the lung cancer risk of the order of 20–50%, while extrapolation, based on risk in active smokers and the relative exposure of passive and active smokers, only indicates an increase of the order of 10%, but they argue this discrepancy is only minor relative to the various uncertainties involved. While the figure of 20–50% seems reasonable (a weighted average relative risk of about 1.3 was cited in the paper), the estimate of 10% seems far too high.

This 10% estimate is stated to be arrived at by linear extrapolation using data on urinary cotinine from a British study (Wald *et al.*, 1984). This study found that whereas cigarette smokers had a median urinary cotinine of 1,645 ng/ml, non-smokers exposed to other people's smoke had a cotinine of 6 ng/ml, i.e. only 0.36% as high. An extrapolated estimate of 10% increase in lung cancer risk in non-smokers would imply about a 25-fold increase in lung cancer risk in smokers. For women, this conflicts sharply with the 3–5 fold increase in lung cancer risk commonly reported in association with active smoking (Surgeon General, 1982). Put the other way round, linear extrapolation using relative urinary cotinine levels would produce a predicted increase in lung cancer risk in passive smokers of 1 to 2%. Quadratic extrapolation would of course produce still lower predictions.

In calculating relative doses of passive and active smokers, estimates based on relative particulate matter retention give much lower values than those based on relative cotinine measurements; the discrepancy between the dosimetry and the epidemiology then becomes even more obvious. Passive smokers take in very small amounts of smoke compared with active smokers and I retain the view expressed in the paper that 'the marked increases in risk noted in some studies are more likely to be a result of bias in the study design than of a true effect of passive smoking'. The doubt as to whether the increased risk of lung cancer seen in non-smoking spouses of smokers is actually a direct result of passive smoking is also stated in a recent

© The Macmillan Press Ltd., 1986

IARC Monograph (IARC, 1986) which pointed to the substantial difficulties in determining passive smoke exposure and the need for further evidence.

Our paper has produced a range of reactions. One lesson to be learnt is how important it is to refer to the original material before forming a proper judgment about a piece of work.

Yours etc.

P.N. Lee,
Independent Consultant in Statistics,
25 Cedar Road, Sutton,
Surrey SM2 5DG, UK

**References**

IARC (1986). *IARC Monographs on the evaluation of the carcinogenic risk of chemicals to humans. Vol. 38, Tobacco Smoking*. World Health Organisation, International Agency for Research on Cancer, Lyon.

LEE, P.N., CHAMBERLAIN, J. & ALDERSON, M.R. (1986). Relationship of passive smoking to risk of lung cancer and other smoking-associated diseases. *Br. J. Cancer*, **54**, 97.

LEE, P.N. (1984). Passive smoking. In *Smoking and the Lung*, Cumming, G. & Bonsignore, G. (eds). Plenum Publishing Corporation.

PETO, J. & DOLL, R. (1986). Passive smoking. *Br. J. Cancer*, **54**, 381.

SURGEON GENERAL (1982). The Health Consequences of Smoking. Cancer. A report of the Surgeon General. U.S. Department of Health and Human Services, Office on Smoking and Health, Rockville, Maryland.

WALD, N.J., BOREHAM, J., BAILEY, A., RITCHIE, C., HADDOW, J.E. & KNIGHT, G. (1984). Urinary cotinine as marker of breathing other people's tobacco smoke. *Lancet*, **i**, 230.